# Theme-Based Block Play Intervention Facilitates Chinese Preschoolers’ Language Development: A Quasi-Experiment

**DOI:** 10.3389/fpsyg.2022.741113

**Published:** 2022-05-24

**Authors:** Liman Cai, Dandan Wu, Jingjing Zhou, Hui Li

**Affiliations:** ^1^School of Education, South China Normal University, Guangzhou, China; ^2^Faculty of Education, The Education University of Hong Kong, Hong Kong, Hong Kong SAR, China; ^3^School of Education, Macquarie University, Sydney, NSW, Australia; ^4^Shanghai Institute of Early Childhood Education, Shanghai Normal University, Shanghai, China

**Keywords:** block play, theme-based activity, playful learning, language development, early language

## Abstract

This study investigated the role of theme-based blocks play in enhancing Chinese children’s language capacity with a quasi-experiment. Altogether 61 young children were assigned to the experiment group (*M*_age_ = 5.83, SD = 0.25, 56.25% girls) and the control group (*M*_age_ = 5.87, SD = 0.28, 51.72% girls). The experiment group was engaged in a 12-week theme-based block play intervention programs, whereas the control group received no interventions but free block play during the parallel time sessions. All the children were tested with the Language Assessment for Preschool Children (LAPC) before and after the intervention. The ANCOVA results indicated that the experimental group significantly improved in LAPC test, whereas the control group showed no significant change. The educational implications of these findings are discussed.

## Introduction

Exposure to block-building activities is often deemed important for children’s early development in various domains, such as cognition, socialization, motor, and fine motor skills, as well as language and literacy (Stroud, 1995; Pickett, 1998; Christakis et al., 2007; Cohen and Uhry, 2007; Hanline et al., 2010; Ferrara et al., 2011). Furthermore, many studies have demonstrated the benefits of block play on visual perception ability, mathematics learning (Pirrone and Di Nuovo, 2014; Trawick-Smith et al., 2017), mental imagery (Pirrone et al., 2015, 2018), and other developmental domains (Verdine et al., 2014). In addition, previous studies have found a significant effect of block play on children’s language development, especially in English-speaking samples (Cohen and Emmons, 2017). Yet, there is a dearth of evidence on the feasibility of block play as a playful learning activity in the early years, especially in the Chinese context. Furthermore, although previous studies have established a link between block play and spatial language (see Yang and Pan, 2021), no study has examined whether and how early interventions on theme-based block play are related to later language development in a school setting. Thus, this study aimed to examine the effect of theme-based block play on Chinese children’s language development to fill the gap.

## Block Play and Language Development in Early Years

Block play is a common activity in the early years, which has also been defined as an open-ended, creative, and valuable play and learning experience available to every setting, offering children enormous opportunities to explore their surrounding world by taking apart and putting back together any block-based creation they can think of (Rybczynski and Troy, 1995; Ferrara et al., 2011; Cai et al., 2020). In the past decades, researchers reached a consensus that block play in the early years generates various kinds of benefits for children’s development, which include but are not limited to: motor and fine-motor skills (Hanline et al., 2001), social development (i.e., peer-relationship, cooperation, prosocial behaviors, etc., see Rybczynski and Troy, 1995), cognitive development [i.e., spatial ability, see Wolfgang et al. (2001) for example; math achievement, see Hanline et al. (2010) for example; engineering potentials, etc., see Cai et al. (2020) for example], and language development (Stroud, 1995; Pickett, 1998; Christakis et al., 2007; Cohen and Uhry, 2007; Ferrara et al., 2011).

In particular, researchers have long found that block play in the early years has laid the foundation for children’s development of language and literacy (Stroud, 1995; Pickett, 1998). During block plays, children can gain plenty of vocabulary and extensive language practice by conceptualizing meanings about their surrounding world and understanding the preliminary literacy activities generated by block plays (Christakis et al., 2007; Cohen and Uhry, 2007; Ferrara et al., 2011). Yang and Pan (2021) explored children’s spatial language use during block play and their associations with the level of block play by observing their daily free play with blocks in the classroom. They found block play can facilitate young children to produce a variety of spatial language, including spatial locations, deictic terms, dimensions, shapes, and so on. Their study also confirmed that the use of spatial language during the block play process had a significant positive correlation with age, the construction structure, and the form of block building. Christakis et al. (2007) experimented with randomized control trial design to examine 220 children aged from 1.5 to 2.5, who found that distributions of blocks in the family led to significant language development progress in children from middle- and low- SES families. Cohen and Uhry (2007) also observed the 5-year-old preschoolers’ conversations in block play and found that children performed different strategies communicating in three types of social relationships in block play (individuals, dyads, and groups), which suggested that block play can be a suitable context for encouraging language development and appropriate teaching activities for language learning in early years. To investigate how block play can influence the variations in spatial language development, Ferrara et al. (2011) assigned children and their parents to three conditions of block activities, including free play, guided play, and play with preassembled structures. They found that both parents and children involved in the guided play produced significantly higher proportions of spatial talk than those in the other two conditions, suggesting the feasibility of conducting simple-to-execute educational interventions. Overall, all these previous studies jointly noticed the correlations between block play and language development, implicating the educational values of using block play to enhance language learning (Christakis et al., 2007; Ferrara et al., 2011). Very recently, Thomson et al. (2020) found that fathers’ spatial language support can facilitate their daughters’ early math skills development during block play, highlighting the advantage of block play as a learning context. However, there is no educational experiment to confirm whether block play can significantly improve language development as a teaching context. Therefore, the current study aims to fill this gap.

## Theme-Based Block Play as a Learning Activity in the Chinese Context

In recent years, the possibility and feasibility of playful learning through block plays have caught much attention from researchers and educators in Chinese preschool education. Specifically, theme-based block play (or theme-based constructive play), an exploratory construction activity carried out by children, has become more common and popular than before in Chinese kindergarten. During a theme-based block play, children are allowed to operate different types of building blocks, using construction skills such as tiling, widening, enclosing, inlaying, and combining their own experience under the guidance and support of teachers (Liu, 2004; Cai, 2018; Yan, 2018; Wang, 2019; Cai et al., 2020). Furthermore, Liu (2004) stated that children could acquire the skills of building blocks and gain various beneficial experiences in the theme-based block building activity, uniting all kinds of developmental domains into one early learning curriculum. In this perspective, block play has been viewed as a comprehensive playful learning activity that integrates aesthetics, creativity, cognition, and language development (Liu, 2004). Under this trend, more and more kindergartens and teachers in China have experienced plenty of teaching practice in organizing theme-based block play activities, gradually establishing a comprehensive and developed program (Yan, 2018; Wang, 2019; Cai et al., 2020). For example, Yan (2018) concluded four elements of theme-based block play activity in early years learning, including (1) life-oriented construction theme to stimulate children to explore and conceptualize, (2) appropriate ways to respect and understand children’s independent exploring and trials, (3) sufficient space to provide proper guidance and encouragement, and (4) diversified and comprehensive, constructive play to enhance children’s playful learning and constructive development. Later, Wang (2019) proposed a three-stage framework for organizing theme-based block play activity in kindergarten, which consists of (1) interest arousing, (2) exploring and constructing, and (3) sharing and evaluation. There are different learning content and guidance tips regarding different developmental objectives in each stage. According to this framework (Wang, 2019), the interest arousing stage aims to intrigue children’s willingness and curiosity to participate in the theme-based block play activity. During this time, the theme of the block play should be established. Then, the stage of exploring and constructing targets stimulates children to think, explore, construct, conceptualize, and enrich their experience and thoughts by meaning-making and the progress of block play. The last stage, sharing and evaluation, enables the children to reflect on their block play process and provides a base for them to gain feedback.

The values of such a theme-based block play have also been advocated in the past decades, especially for providing considerable and potential opportunities for early language development (Heisner, 2005; Cai, 2018). Heisner (2005) believes that using classroom toys (building blocks) to simulate story themes is a simple way for preschool teachers to consolidate new vocabulary and concepts, effectively helping children better understand story content. In particular, Children can develop dialogue and practice language when playing games through a familiar. Coincidently, Cai (2018) conducted an experimental study and found that block play played a noticeable role in promoting preschool children’s expressive language ability but not preschool children’s comprehensive language ability. They further argued that this was because, during the intervention period, most of the block play activities in their study were limited in single dimension constructive play without a comprehensive theme. As such, they inferred that theme-based block play might have more advantages in improving children’s language capacity. Although the three-stage framework for theme-based block play (Wang, 2019) has been adopted in more and more Chinese kindergartens, to our best knowledge, there is no existing study examining the effect of such a learning program on children’s language development. This study aims to examine the effectiveness of the theme-based block play being a language intervention program to fill this gap. Accordingly, the following question and hypotheses guided this study:

Is the theme-based block play intervention program effective in improving Chinese preschoolers’ language capacity?

*Hypothesis 1*: The experimental and the control groups should have no significant differences in the pretest of LAPC before the theme-based block play intervention program.

*Hypothesis 2*: The experimental group would significantly outperform the control group in the posttest of LAPC after the theme-based block play intervention program.

## Materials and Methods

### Participants

One public kindergarten in Guangzhou, Guangdong Province, consented to participate in this study, from which two Senior classes were sampled to participate in the intervention. Altogether 61 children (Mean Age = 5.85) participated in this study and were assigned to the condition, with one class as the experiment group (*n* = 32) and the other class served as the control group (*n* = 29). The two groups did not differ significantly in age, gender, and index of LAPC (Table 1). All the 61 participating children from middle SES families lived in the neighborhood with their parents in Yuexiu District, where the GDP ranked top three in Guangzhou city (Guangzhou Government Annual Report, 2021). A *post hoc* power analysis was conducted on G*Power 3.1^1^ to calculate the power of this comparison between two independent group means, using a two-tailed test, a large effect size (Cohen’s *d* = 0.9; Cohen, 1992), and an alpha of 0.05. The result showed that the power of the test was 0.93, which is satisfactory in this study. Furthermore, the children in both groups were not diagnosed with any developmental delay or cognitive difficulties.

**Table 1:** Descriptive statistics.

	Experiment Group (*n* = 32)	Control Group (*n* = 29)		
Gender: Boy	14	14		
Gender: Girl	18	15		
Age	5.83	5.87		
Age range	5.35–6.25	5.38–6.33		
	*M (*SD*)*	*M (*SD*)*	*t*	*sig.*
Listening and Speaking	26.81 (7.20)	28.76 (5.49)	1.178	0.244
Reading and Storytelling	10.97 (5.49)	12.79 (3.40)	1.542	0.128
Total LAPC score	37.78 (10.38)	41.55 (7.36)	1.622	0.110

LAPC, Language Assessment for Preschool Children (Scores of pre-test).

The independent *t*-test showed no significant difference between the two groups on the assessments in this study. Both groups of children shared the same kindergarten curriculum and experienced the same daily routine and outdoor activities in the same kindergarten. Both participating groups were blind to this experimental condition and hypothesis. All the participating children have former experiences in block play activities. In China, each class has one class teacher (班主任) and two assistant teachers (助教), plus a nursemaid to take care of the cleaning, feeding, and sleeping matters. Generally, the class teacher is in charge of the teaching and learning activities, with the help of two assistant teachers. The two class teachers of both groups had no significant differences in qualifications and teaching experiences. Specifically, the class teacher of the experiment group had 4.5 years of teaching experience and a Bachelor of Education (B. Ed.). Also, with BEd, the control group class teacher had taught in the kindergarten for 2 years. The teacher of the experiment group had rich experience in organizing block-related activities in the early years and had been well trained for 2 years to conduct the block-building intervention program. The class teacher of the control group had no experience in block activities. The experiment group teacher did not receive any other training alongside the block building ones during the intervention period.

### Instrument

Children’s language ability was tested using the Language Assessment for Preschool Children (LAPC, Language Centre in Tianjin Normal University, 2013) before and after the intervention. The children’s group was not disclosed or labeled when administering LAPC to assure the examiner’s blindness to the condition and hypothesis. The LAPC consists of two tasks: (1) Listening and Speaking and (2) Reading and Storytelling. The tasks and the whole LAPC demonstrated an excellent internal reliability: Cronbach’s alpha_(1)_ = 0.802; Cronbach’s alpha_(2)_ = 0.901; Cronbach’s alpha _LAPC_ = 0.742. And the inter-rater reliability for Listening and Speaking, Reading and Storytelling, and whole LAPC is 0.882, 0.884, and 0.868, respectively, which is also acceptable.

#### Listening and Speaking

This task was designed to test children’s capacity in listening and speaking. In this task, the child was invited to listen to the story (see Appendix 1) narrated by the examiner and answer the related questions afterward. Altogether there were eight questions in this task: *(1) what is Miao (the story’s subject) doing on this day? (2) How did Miao fly to the sky? (3) why was Miao able to fly in the sky? (4) What did Miao see when she was flying in the sky? (5) What happened or what had Miao done when she was flying in the sky? (6) Where did Miao end up flying? (7) who did Miao see on the roof of the house? What did this person do? (8) Where did Miao jump off?* For each question, the child scored 0 if the child cannot reach the standard, 3 if the child gets close to the standard, and 5 if the child reaches the standard completely. The total score for this task ranged from 0 to 40. Two researchers evaluated each child simultaneously, and the mean of their scores was calculated and analyzed in this study. In this case, there was no need to check the interrater reliability.

#### Reading and Storytelling

This task aimed to observe the child’s capacity in reading and storytelling. In this task, the child was asked to read a picture book and then tell its story (see Appendix 1). Accordingly, five aspects of language capacity were observed, including *(1) vocabulary:* correctly use verbs, nouns, adjectives; *(2) sentence complexity:* correctly use a long sentence with turning structure or adverbial clause; *(3) utterance length:* each sentence has three or above words that are correct and appropriate; *(4) temporal aspect:* correctly use temporal aspect, such as ‘just then’, ‘suddenly’ and so forth; *(5) narrative integrity:* tell the story clearly, coherently, and completely. For each aspect, the child scored 0 if they could not reach the standard, 3 if they got close to the standard, and 5 if they reached the standard completely. Thus, the total score for this task ranged from 0 to 25. Two researchers evaluated each child simultaneously in this test, and the mean of their scores was adopted in this study; thus, there was no need to check the interrater reliability.

### Procedure

#### Ethical Clearance

This study was reviewed and approved by the first author’s university. Written consent forms were gained from the participating kindergarten, the principal, the class teachers, and the parents to participate in this study. All the parents were briefed about this study and consented to allow their children to participate. The participating young children were introduced to the task and verbally agreed to attend this study. They were allowed to deny evaluation or withdraw from this study at any time.

#### Intervention Program: Theme-Based Block Play

The theme-based block play intervention program was designed based on the three-stage framework by Wang (2019). Specifically, for each activity, there were three sections, including (1) interest arousing introduction; (2) exploring and constructing; (3) sharing and evaluation (for details, please refer to Appendix 2). Wang (2019) mentioned that this three-stage framework was designed to raise children’s interests and potential to enhance their language development. The specific intervention begins 1 month after the beginning of the school year for young children. The intervention program was conducted in the construction room within the kindergarten, twice a week, with 1 h each time, generating 12 weeks of intervention. Before the intervention of theme-based building block activities, the experiment group’s class teacher and assistant teachers were introduced and trained about the intervention scheme, teaching skills, evaluation methods, and the implementation process of theme-based block play activities. During the intervention of theme-based block play activities, the children in the experimental group participated in three stages of three different themes, namely “Journey to the Zoo,” “Robot Story,” and “On the Road.” Each stage involved eight times of block play activities, resulting in 24 times altogether. Each activity was guided by the class teacher, who had rich experience in early theme-based block-building activities. Meanwhile, the control group was given independent block play without teacher intervention. In particular, the theme-based block activities were initiated and led by the class teacher, who would introduce the theme and the relevant block activities. This was conducted in the whole class mode. Then, the children would be grouped and guided to discuss the possible building projects for their groups. After the discussion, they would do building blocks for their projects. Again, this should be delivered in group mode, with the help and guidance provided by the class teacher and assistant teachers.

## Results

### Testing Hypothesis 1

To examine whether the experiment and the control group significantly differed in the pretest (Hypothesis 1), a set of paired-samples *t*-tests on the scores of all LAPC tasks were conducted. As shown in Table 2, in the Listening and Speaking task, no significant differences were found between the experiment group (*M* = 26.81, SD = 7.20) and the control group (*M* = 28.76, SD = 5.49), *t* = 1.178, *p* > 0.05, Cohen’s *d* = 0.305. In the Reading and Storytelling task, no significant differences were indicated between the experiment group (*M* = 10.97, SD = 5.49) and the control group (*M* = 12.79, SD = 3.40), *t* = 1.542, *p* > 0.05, Cohen’s *d* = 0.399. In the total LAPC task, there was no significant differences found between the experiment group (*M* = 37.78, SD = 10.38) and the control group (*M* = 41.55, SD = 7.35), *t* = 1.622, *p* > 0.05, Cohen’s *d* = 0.419. All these *t*-test results indicated no significant differences between the experiment and control groups, providing empirical support for hypothesis 1.

**Table 2:** Means, SDs, and *t*-tests results for the LAPC tasks in pretest and posttest (Experiment group: *n* = 32; Control group: *n* = 29).

	Pretest		Posttest		Gain scores		*T*-test	sig.
	Experiment	Control	Experiment	Control	Experiment	Control		
Listening and Speaking	26.81 (7.20)	28.76 (5.49)	32.31 (3.77)	30.76 (4.73)	5.50 (5.13)	2.00 (5.37)	−2.60	0.01
Reading and Storytelling	10.97 (5.49)	12.79 (3.40)	17.13 (3.63)	13.28 (2.25)	6.16 (4.98)	0.48 (3.91)	−4.91	0.00
Total LAPC	37.78 (10.38)	41.55 (7.35)	49.44 (6.26)	44.03 (4.86)	11.66 (7.55)	2.48 (7.32)	−4.81	0.00

### Testing Hypothesis 2

To confirm whether the experiment and the control group significantly differed in the posttest (Hypothesis 2) while controlling for pretest scores, a set of one-way ANCOVAs was conducted. Levene’s tests and normality checks were carried out, and the assumptions were met. Altogether, there was significant difference in mean score increase of the overall LAPC tasks between the control group (*M* = 44.03, SD = 4.86), and the experiment group (*M* = 49.44, SD = 6.26), *F* (1, 58) = 30.503, *p* < 0.001, $\eta_{p}^{2}$ = 0.345. In particular, in the Listening and Speaking task, a significant difference was found between the control group (*M* = 30.76, SD = 4.73) and the experiment group (*M* = 32.31, SD = 3.77), *F* (1, 58) = 6.531, *p* = 0.013 < 0.05, $\eta_{p}^{2}$ = 0.101. In the Reading and Storytelling task, there was a significant difference between the control group (*M* = 13.28, SD = 2.25) and the experiment group (*M* = 17.12, SD = 3.63), *F* (1, 58) = 32.814, *p* = <0.001, $\eta_{p}^{2}$ = 0.361. In sum, all the ANCOVA tests indicated significant differences between the experiment and control groups while controlling the pretest language scores, providing empirical evidence to partially support Hypothesis 2. However, according to Cohen (1992), the effect size in this comparison should be regarded as ‘small’, as they ranged between 0.10 and 0.37.

### Testing the Group Differences in Gain Scores

**Figure 1:**
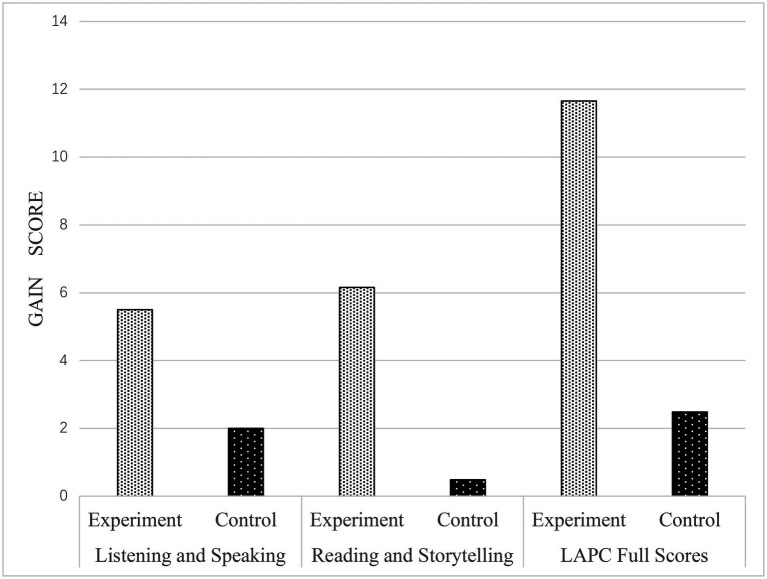
Comparison of gain scores in the LAPC tasks between the experiment and control groups. Experiment group: *n* = 32; Control group: *n* = 29.

Last, we calculated the gain scores between the posttest and pretest for each child. As shown in Table 2, in the Listening and Speaking task, significant differences were found in the gain scores between the experiment group (*M* = 5.50, SD = 5.13) and the control group (*M* = 2.00, SD = 5.37), *t* = − 2.60, *p* < 0.05, Cohen’s *d* = 0.666. In the Reading and Storytelling task, significant differences were found in the gain scores between the experiment group (*M* = 6.16, SD = 4.98) and the control group (*M* = 0.48, SD = 3.92), *t* = − 4.91, *p* < 0.001, Cohen’s d = 1. 267. In the whole LAPC task, significant differences were found in the gain scores between the experiment group (M = 11.66, SD = 7.55) and the control group (*M* = 2.48, SD = 7.32), *t* = − 4.81, *p* < 0.001, Cohen’s d = 1.235. According to Cohen (1992), the effect size in this comparison should be regarded as ‘medium to large’, as they ranged between 0.60 and 1.27. All these *t*-test results jointly indicated significant differences in gain scores between the experiment and control groups (Figure 1).

## Discussion

This study is a preliminary exploration of the impact of theme-based block activities on young children’s language capacity in China. The significant results might not necessarily provide sound evidence of the ‘training effect’ with a quasi-experiment. However, the findings might have implications for teaching practice and future studies that will be addressed in the following sections.

### The Impact of Theme-Based Block Play on Early Language Development

This study found that the experimental group outperformed the control group in the posttest and the gain scores. This result provided empirical evidence demonstrating the effect of theme-based block play on enhancing children’s language development, including both expressive and comprehensive domains. This finding is consistent with the previous studies, which jointly noticed that block play has benefits in promoting early language development (Christakis et al., 2007; Cohen and Uhry, 2007; Ferrara et al., 2011). However, this finding provides different evidence about the effect of block play on language development from Cai (2018), who found that single-dimension block play can only improve preschool children’s expressive language ability, but not their comprehensive language ability. This discrepancy might be caused by the fact that compared with the theme-based block play, the single dimension block play may be less effective in stimulating children’s thinking, negotiating, and discussion on such as goal-oriented plans, thereby reducing children’s opportunities of planning game plots, experiencing role relationships and talking about playful events (Wang, 2019). In other words, without a theme-based context to organically incorporate all these elements, the effect of block play on early language development would be limited (Cai, 2018). Therefore, we would not regard this finding as inconsistent with Cai (2018); instead, we would take it as a piece of supplementary evidence to understand the educational values of block play instead.

So, why could theme-based block play improve children’s language capacity in both expressive and comprehensive domains? First of all, the teacher created a learning context by posing a question to guide the theme of this activity. For example, to initiate the theme-based block play activity ‘Journey to the Zoo’, the class teachers asked children the leading question: “How can I help Little Pierre go to the zoo?” In this process, the children were allowed to express their thoughts and feelings actively, and even to propose their suggestions to solve the problem, such as “let him play something else first,” “we build a zoo for him,” “the zoo is far away, we need transportation first,” and so forth. Second, as the theme was generated from children’s interest, they had greater enthusiasm for participation and were stimulated by the problem situation to maintain their motivation, which provided the opportunity to develop their language ability, their willingness and attention to listen, their desire to express, and thereby enlarging the learning chances. Third, as the theme was not prescribed by teachers, and the children were willing to accept it as their own ideas, the equality of communication between teachers and children was established, maximizing the guiding role of the teachers. Eventually, from “Little Pierre wants to go to the zoo” to “Invite Little Pierre to our zoo,” the themes were developed and evolved throughout the block play activities, thereby stimulating children to think and talk in real situations.

### Implications for Educational Practice and Future Studies

These findings have implications for educational practice in early childhood settings and teacher training institutions. First, the preservice early childhood teacher education programs should include teaching skills and theories about block play, which is indirectly but eventually beneficial to young children’s cognitive and language development. Second, the teacher professional development activities should also include the block play skills to equip all the inservice teachers with rich experience and skillful practice in block play. This will also help them implement effective theme-based block play programs, significantly promoting young children’s cognitive and language development.

These findings also have implications for future studies on early childhood block-building activities. Unfortunately, the research design (quasi-experiment) and the associated limitations (as reviewed in the next section) have prevented this study from generating sound evidence to support the training effect. However, the association between theme-based block play activities and young children’s language development has been confirmed in this study. Further studies with large sample sizes or randomized free trials should be conducted to confirm the cause-effect relationship between the theme-based block building activity and young children’s cognitive and language development.

## Conclusion and Limitations

As the first empirical examination of the effect of theme-based block play activity on Chinese children’s early language capacity, this study developed, implemented, and evaluated a set of theme-based block play intervention activities. The results found no significant differences between the experimental and control groups in the pretest but significant differences in the posttest and the gain scores, supporting hypotheses 1 and 2. Overall, this study confirmed the impact of theme-based block play on young children’s language development.

This study has some limitations that could be addressed in future studies. First, this study’s sample size is limited, even though the effect size for the gain scores comparison ranged from medium to large. More participants with diversified backgrounds and expanded age ranges could be included in future studies. Second, this quasi-experiment did not randomly assign the children to the experiment and control conditions and did not control for general parent–child interaction with and without block play at home. Future studies should consider the possible confounding factors such as the home language environment and home block building experiences. Third, there could have been other differences in teaching quality between the experiment and control groups beyond the block play activities; for instance, the class teachers for the two groups differed in teaching experience, thus might have contributed to the differences in the relevant assessments.

## Data Availability Statement

According to the ethical clearance issued by SCNU, the data presented in this study are not publicly available as they include private information about the participating children and teachers. Data will be made available on request from the first author.

## Ethics Statement

The studies involving human participants were reviewed and approved by South China Normal University. Written informed consent to participate in this study was provided by the participants’ legal guardian/next of kin.

## Author Contributions

LC designed and administered the whole study. LC and JZ collected and managed the data under the supervision of HL. DW and JZ analysed the data and drafted the manuscript under the supervision of HL. All authors contributed to the article and approved the submitted version.

## Funding

This study was fully funded by the 2016 National Education and Science Key Fund (DBA160251) for LC.

## Conflict of Interest

The authors declare that the research was conducted in the absence of any commercial or financial relationships that could be construed as a potential conflict of interest.

## Publisher’s Note

All claims expressed in this article are solely those of the authors and do not necessarily represent those of their affiliated organizations, or those of the publisher, the editors and the reviewers. Any product that may be evaluated in this article, or claim that may be made by its manufacturer, is not guaranteed or endorsed by the publisher.

## Footnotes

^1^ https://www.psychologie.hhu.de
