# Supplementary material for: Theme-Based Block Play Intervention Facilitates Chinese Preschoolers’ Language Development: A Quasi-Experiment
Source: Front Psychol. 2022 May 24;13:741113. doi: 10.3389/fpsyg.2022.741113 (PMC9171375; doi:10.3389/fpsyg.2022.741113)
Supplement: Supplementary file 1 [file Presentation_1.pdf]

Appendix 1. Language Assessment for Preschool Children (Protocol)

Part A. Listening and Speaking

1. Listen to the Story:

One day, Miao was walking along the road, holding up an umbrella. Suddenly, a strong wind blew up and blew Miao into the sky. Miao floated up in the sky with an umbrella. During her floating in the sky, Miao saw a green grassland on the ground with several children playing football on it. When Miao continued to fly in the sky, a wild goose flew past her side, and Miao sent warm greetings. After a while, Miao landed on the roof of a house. Just below the house, Miao saw her Mom in the yard. Then, Miao jumped down from the roof. Fortunately, Miao was caught safely by her Mom.

2. Answer the Questions:

(1) what is Miao (the subject of the story) doing on this day?
(2) How did Miao fly to the sky?
(3) why was Miao able to fly in the sky?
(4) What did Miao see when flying in the sky?
(5) What happened or had Miao done when flying in the sky?
(6) Where did Miao end up flying?
(7) who did Miao see on the roof of the house? What did this person do?
(8) Where did Miao jump off?

3. Scoring:

**Part A. Scoring standard**	**Scored**
If the child answered the question incorrectly or was unable to justify the answer;	0
If the child's answers were basically correct, with a minor mistake (e.g., grammar)	3
If the child answered and explained correctly.	5

Note. The total score for this part ranged from 0 to 40.

Part B. Reading and Storytelling

1. Read the picture book and then tell the story

Story brief:

"One day, a little rabbit was walking on the road with a basket in her hand. Suddenly, she saw an apple tree in the distance. The little rabbit wanted to eat apples very much. So, she climbed up the apple tree and put all the picked apples in the basket. There were too many apples in the basket, which broke the branches. As a result, the little rabbit fell from the tree. The little rabbit fell to the ground and was very unhappy. The apples rolled all over the floor. Just then, a little hedgehog happened to pass by. He helped the little rabbit pick up all the apples on the ground and put them in the basket. The little rabbit smiled happily. "

2. Observation:

In this task, the child was asked to read a picture book and then tell its story.

A totally five aspects of language capacity were observed, including:

(1) vocabulary: correctly use verbs, nouns, adjectives;

(2) sentence complexity: correctly use a long sentence with turning structure of adverbial clause;

(3) utterance length: each sentence has three or above words that are correct and appropriate;

(4) temporal aspect: correctly use temporal aspect, such as ‘suddenly’, ‘just then’ / ‘at this moment’ and so forth;

(5) narrative integrity: tell the story clearly, coherently, and completely.

3. Scoring:

Part B. Scoring standard	Scored
If the child cannot reach the standard;	0
If the child gets close to the standard;	3
If the child reaches the standard completely.	5

Note. The total score for this part ranged from 0 to 25.

Appendix 2. Three-stage Framework for Theme-based Block Play (Wang, 2019)

**Three-stage Flow**	**Key Foci of Each Stage**
Stage 1. Arousing Interest	This stage is crucial to arouse children's interest through effective observation, targeted dialogue, and discussion to discover the relevant theme related to children's daily lives. The theme should be valuable for children's current or future development, further stimulating children's interest in construction and creating a discussion atmosphere. The focus of this stage is to help children make clear the purpose of construction, triggering children's planning and thinking and stimulating children's desire to express an interest in activities.
Stage 2. Exploring and Constructing	This stage focuses on facilitating children's thinking, constantly enriching and perfecting their ideas, and promoting the development of playful learning. Children's active communication and exchange are triggered in-group cooperation by observing the works (such as pictures, demonstrations, etc.). Children's thinking leads to group negotiation and discussion, thereby constantly solving problems, eventually promoting the construction activities and completing the construction.
Stage 3. Sharing and Evaluation	This stage mainly focuses on reviewing children's works to form interesting and playful situations and provoke their willingness to express thoughts and feelings and share with their peers.

Example of Theme-based Block Play Activity Plans

<table>
<tr><th>Theme(s)</th><th>Stage(s)</th><th>Block Play Content</th></tr>
<tr><td rowspan="3">Journey to the Zoo
(9 Activities)</td><td>Arousing Interest</td><td>1. Context introduction: Storytelling
2. Group discussion： to design a zoo
3. Task allocation: planning and arranging</td></tr>
<tr><td>Exploring and Constructing</td><td>This stage involves three sections based on the task allocation: building the animals, the zoo, and the necessary equipment. For this stage, there are four steps：
1. Confirm the goal and planning
2. Guided block building
3. Sharing the problems and brainstorming
4. Review and plan the next step</td></tr>
<tr><td>Sharing and Evaluation</td><td>1. Election of tour guide for visiting the zoo
2. Contextual play: children play the role of tour guide to lead the main character in the story to visit the zoo, which is built by the children in the last stage.
3. Dramatic play: the main character leads the children to visit the zoo.
4. Review and Evaluation</td></tr>
<tr><td rowspan="2">Robot Story
(7 Activities)</td><td>Arousing Interest</td><td>1. Interest arousing: the children were found to be interested in the gear materials (in the last activity)
2. Context introduction: Robot contest is coming soon
3. Creation (art/drawing): design the robot</td></tr>
<tr><td>Exploring and Constructing</td><td>Based on the previous robot design, the children were grouped and cooperated to build the robot. They equipped the robot with a charging station and related facilitations. For this stage, it includes：
1. Planning: confirm and allocate the construction task
2. Free block building: group cooperation with the guidance of teachers
3. Sharing the problems and solutions during the construction process</td></tr>
</table>

		4. Improving the robot and its station in the next activity
	Sharing and Evaluation	1. Robot Contest: election of the most population group<br>2. Review and Evaluation
**On the Road** (7 Activities)	Arousing Interest	1. Interest arousing: recently, the theme of the classroom environment creation* is about the 'car', which intrigued children a lot about the transportation things in life.<br>2. Group discussion: what is there on the road? Preparing to build a road in the block corner in the classroom.<br>3. Task allocation: planning and arranging
	Exploring and Constructing	Based on the previous group discussion and task allocation, the children were assigned as 'vehicle group', 'transportation facilities (i.e., bus station, traffic light)', 'construction group', 'pedestrian bridge group', etc.<br>For this stage, basic steps include：<br>1. Planning: confirm and allocate the construction task<br>2. Free block building: group cooperation with the guidance of teachers<br>3. Sharing the problems and solutions during the construction process<br>4. Improving the building works in the next activity
	Sharing and Evaluation	1. Contextual play: Within the block-building transportation built by the children, they are invited to attend the role play with the theme of 'traffic light.'<br>2. Discussion of the traffic rules: safety education<br>3. Review and evaluation.

Note. Environment creation*: 'environment creation' is popular in Chinese kindergarten (mentioned as '环境创设／环创'), which refers to providing rich, related, and appropriate materials in the learning environment (within the classroom) where children stay. Generally, the materials in the environment creation are replaced regularly (i.e., once a week/month), usually with a comprehensive, united, and meaningful theme.
